# Unmet Needs and Challenges in Cancer-Associated Venous Thromboembolism

**DOI:** 10.3390/ijms27041756

**Published:** 2026-02-12

**Authors:** Sanober Nusrat, Sayeed Khan, Kisha Beg, Gary Raskob

**Affiliations:** 1 Hematology-Oncology Section, Department of Internal Medicine, College of Medicine, University of Oklahoma Health Campus, Oklahoma City, OK 73104, USA; 2 Department of Molecular and Cellular Physiology, Albany Medical College, Albany, NY 12208, USA; khans16@amc.edu; 3 Hematology-Oncology Section, Department of Pediatrics, College of Medicine, University of Oklahoma Health Campus, Oklahoma City, OK 73104, USA; 4 University of Oklahoma Health Campus, Oklahoma City, OK 73104, USA

**Keywords:** cancer-associated venous thromboembolism, CAT, Khorana score, anticoagulation

## Abstract

Cancer-associated venous thromboembolism (CA-VTE) is a significant complication in oncology, contributing to morbidity, mortality, and increased healthcare utilization. Due to multiple patient- and disease-related factors, patients with cancer are at a markedly elevated risk for VTE, particularly within the first 6 months of diagnosis. The aim of this review is to provide an overview of current challenges and unmet needs in CA-VTE prediction, prevention and management. While the Khorana score remains the most widely used risk stratification tool, its limited sensitivity has prompted the development of more refined models such as PROTECHT, CONKO, ONKOTEV, Vienna-CATS, and COMPASS-CAT. These models incorporate additional clinical variables including cancer subtype, systemic therapies, comorbidities, and emerging biomarkers. However important gaps remain, particularly in addressing bleeding risk, underrepresented racial/ethnic groups, and adapting to novel cancer therapeutics. Recent clinical trials (AVERT, CASSINI) have supported the use of direct oral anticoagulants (DOACs) for primary and secondary prophylaxis in select high-risk populations. However, anticoagulation strategies in complex populations, including those with thrombocytopenia, brain tumors, or concurrent antiplatelet therapy, remain areas of active investigation. Future directions include the integration of genomics, proteomics, and machine learning into risk modeling to enable precision medicine approaches. Ongoing clinical trials are testing the promise of safer prophylactic and therapeutic strategies. Personalized risk assessment and treatment of CA-VTE remain essential to improving patient outcomes in oncology. By consolidating existing evidence and identifying key unmet needs, this review seeks to guide more personalized and effective management of CA-VTE.

## 1. Introduction

Patients with cancer have a high risk of developing venous thromboembolism, described as cancer-associated venous thromboembolism (CA-VTE). In the first 6 months following cancer diagnosis, the risk for VTE is reported to be 12-fold greater than VTE in the general population [1]. According to multiple population- and registry-based studies, 17–29% of the total VTE cases are associated with cancer [2,3,4]. Cohen et al. reported an incidence of 5.8 per 100 person-years in cancer patients for the occurrence of the first CA-VTE episode [5]. Raskob and colleagues reported a population-based study in which the age-adjusted incidence of CA-VTE was 70 per 100,000 adults in the general population, translating to an estimated annual number of 179,000 new cases among adults in the US population (2020) [5,6].

The incidence of CA-VTE seems to be increasing over time. In a California cohort of cancer patients, the cumulative incidence of CA-VTE in pancreatic cancer patients rose from 8.92% during the 2005–2007 era to 11.9% during the 2014–2017 era [7]. Similar incidence rates have also been found in patients with lung cancer and breast cancer [7]. The increasing incidence over time may be attributed to improved diagnosis with high-resolution imaging for cancer staging and response assessments, more appropriate ICD coding, and the introduction of anti-angiogenic agents, tyrosine kinase inhibitors and immunomodulatory drugs [7].

The rate of VTE recurrence in patients with CA-VTE is high and reported at 9.6 per 100 person-years [5]. Patients with pancreatic, ovarian, brain, or lung cancer, and those with advanced stage of cancer are at the highest risk of VTE events, with pulmonary embolism being the most common. After discontinuing anticoagulant therapy, the risk of recurrent VTE among patients with CA-VTE is 10 to 15% during the first six months. Recurrences reach 31% at 2 years and stabilize between 31 and 35% between 2 and 5 years [8].

To provide individualized care for patients with CA-VTE, it is essential to begin with the underlying risk determinants. Patient characteristics, cancer subtypes, and treatment exposures create the biological and clinical foundation upon which thrombotic events arise. These factors not only explain the heterogeneity in VTE incidence across oncology populations but also serve as the building blocks for risk prediction models. By first characterizing these risks, we can then critically evaluate the tools developed to quantify them, highlight their limitations, and outline emerging strategies—including novel biomarkers, computational approaches, and advances in prevention and treatment—that aim to improve outcomes for patients with cancer.

## 2. Risk Categorization (Factors Leading to CA-VTE)

**Figure 1:**
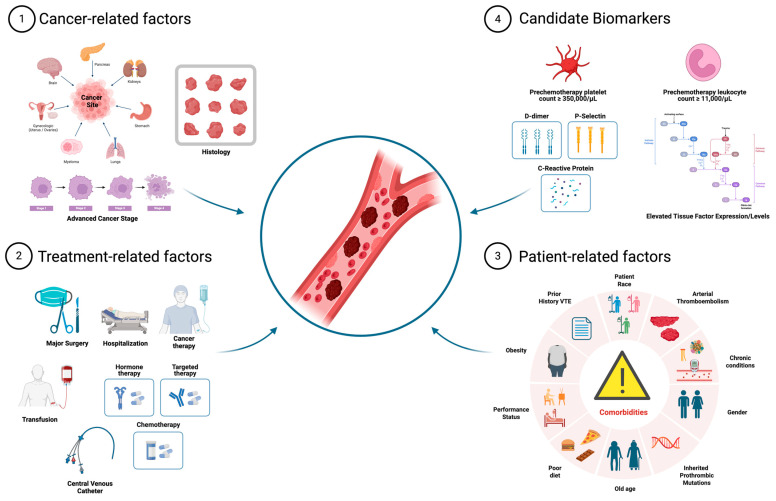
Risk factors and candidate biomarkers for patients at risk for cancer-associated VTE. These four categories are used across risk models to stratify patients at risk for VTE [12]. Created in BioRender. Khan, S. (2026) https://BioRender.com/xbpzvth (accessed on 22 January 2026).

The incidence of CA-VTE is contingent upon multiple variables, such as patient characteristics and preexisting comorbidities, cancer subtype and cancer-directed systemic treatment (Figure 1) [7,9,10,11] Given that anticoagulant exposure causes an increased risk of bleeding, accurate assessment of bleeding and thrombotic risk is needed to assist clinicians in making individualized treatment decisions.

### 2.1. Patient Characteristics

Age, gender and ethnicity have been studied in association with risk for CA-VTE. Patients older than 65 have been shown to have a slightly increased risk of VTE in a retrospective hospitalized cohort with an odds ratio of 1.1 (*p*-value significant) [13]; older patients (≥60 years) undergoing surgery are particularly at a much higher risk with a hazard ratio of 2.63 (95% CI, 1.2–5.7) [14]. However, age is not a significant risk factor among ambulatory cancer patients with good performance status [15].

There are contradictory reports for gender and its association with CA-VTE. While some studies indicate that sex is not a significant factor for CA-VTE [10], a study of all hospitalized patients reported that women have an overall increased risk of VTE [13]. This gender-related increase in VTE may not apply universally across all cancer subtypes. When assessed on its relation to cancer subtypes, a retrospective observational study reported women to have a lower risk for CA-VTE when compared to men for breast cancer (HR 0.67, 95% CI 0.54–0.83, *p* < 0.0001), bladder cancer (HR 0.89, 95% CI 0.80–0.98, *p* < 0.0199) and brain cancer HR 0.82, 95% CI, 0.75–0.90, *p* < 0.0001) [7].

Furthermore, studies have indicated a potential link between the incidence of VTE and race, where African Americans have the highest risk of CA-VTE and Asians/Pacific Islanders have the lowest risk [6,7,16,17]. African American patients also had a significantly higher risk of recurrent CA-VTE compared to White patients in breast (HR, 1.36; 95% CI, 1.08–1.72), prostate (HR, 1.37; 95% CI, 1.09–1.72), and pancreatic cancers (HR, 1.37; 95% CI, 1.01–1.86). Asians/Pacific Islanders and Hispanics, however, did not have significant differences from White patients for recurrent CA-VTE risk [17].

Other patient characteristics including BMI and the number of comorbidities at diagnosis are also associated with risk of CA-VTE. For instance, cancer patients with medical comorbidities, such as infection (odds ratio [OR] = 1.28), pulmonary disease (OR = 1.57), renal disease (OR = 1.41), and obesity (OR = 1.52), have an increased risk of CA-VTE [18]. Personal history of VTE increases the risk of developing CA-VTE by approximately seven-fold [18].

### 2.2. Cancer Subtype and Treatments

**Figure 2:**
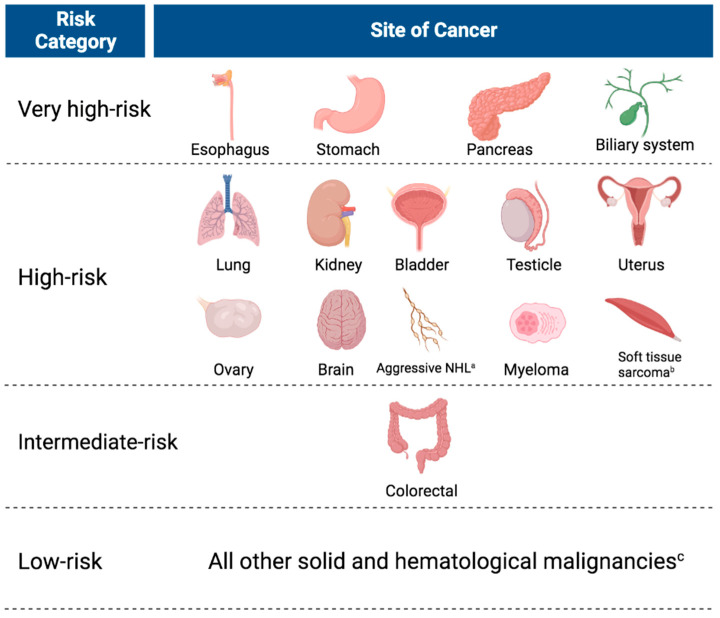
The modified four-tier cancer subtype classification by Li et al., 2023 [20]. Created in BioRender. Nusrat, S. (2026) https://BioRender.com/mwhngg0 (accessed on 29 December 2025). ^a^ Aggressive NHL includes diffuse large B-cell lymphoma, Burkitt lymphoma, acute lymphoblastic lymphoma, and T/natural killer cell lymphoma. ^b^ This category excludes gastrointestinal stromal tumor or Kaposi sarcoma. ^c^ This category includes breast, prostate, head and neck, liver, anal, cervical, acute/chronic leukemia, myelodysplasia, indolent NHL, Hodgkin lymphoma, melanoma, gastrointestinal stromal tumor, Kaposi sarcoma, neuroendocrine, thyroid, and others.

Type of cancer has an independent association with the risk of VTE [19]. It is important to recognize that different cancer subtypes of a specific organ system may also have different risks of thrombosis. Li et al. revised four categories of cancer subtypes based on their risk of thrombosis: (1) a very high-risk group—cholangiocarcinoma/gallbladder cancer added to traditional Khorana score cancer subtypes (esophageal/gastric and pancreatic); (2) a modified high-risk group—aggressive non-Hodgkin lymphoma instead of all lymphoma, ovarian and uterine cancer instead of all gynecologic cancers, myeloma, brain cancer, and soft tissue sarcoma along with lung, bladder, kidney, and testicular cancers; (3) a new risk category, intermediate, for colorectal cancer; (4) the remaining solid and hematologic cancers were grouped as low-risk (Figure 2) [20].

Multiple treatment-related factors produce a proinflammatory state which can increase risk for CA-VTE. These include anticancer drugs, hospitalization, surgery and central venous catheters. Specific systemic anticancer drugs, such as platinums, anthracyclines, antihormonal and immunomodulatory agents, have historically been linked to a higher risk of VTE [21,22,23]. In cases of Cisplatin-based chemotherapy, patients have increased incidence of VTE (1.92% (95% CI, 1.07 to 2.76), 0.79% (95% CI, 0.45 to 1.13), in addition to significantly increased risk of VTEs (RR, 1.67; 95% CI, 1.25 to 2.23; *p* = 0.01) [21]. Furthermore, in newly diagnosed multiple myeloma (MM) patients receiving induction chemotherapy, the addition of thalidomide to a multiagent regimen (including dexamethasone and doxorubicin) significantly increased the incidence of deep vein thrombosis (DVT) from 4% to 28% (*p* = 0.002), with all events occurring within the first three cycles of treatment; this corresponded to a markedly elevated risk of DVT (odds ratio = 9.33, 95% CI not reported) [23]. Furthermore, Zangari et al. also reported that the presence of the doxorubicin regimen in MM treatment increased the cumulative incidence of VTE from 2.6% to 11.6%, and significantly increased the risk of DVT/VTE (*p*  =  0.01, odds ratio  =  11.3) [22].

The advent of newer targeted agents and immune check point inhibitors can cause a treatment-induced hypercoagulable state. The most notable classes of drugs increasing the risk of VTE include epidermal growth factor receptor (EGFR) antibodies (relative risk 1.32–1.58) [24,25] and cyclin-dependent kinase (CDK) inhibitors, especially abemaciclib (relative risk 2.62) [26]. EGFR antibodies, such as cetuximab, trigger the complement-mediated lysis (CML) of tumor cells through the alternative pathway, releasing anaphylatoxins (C3a, C4a, and C5a) and thus, enhancing platelet activation and aggregation [27]. Similarly, CDK inhibitors, such as abemaciclib, induce an increase in the platelet function and coagulation factors, elevating the risk of CA-VTE in breast cancer patients [28]. While vascular endothelial growth factor-targeted therapies and receptor tyrosine kinase inhibitors have been associated with increased risk of arterial thromboembolism, the risk of VTE is not clearly established [29,30,31,32,33]. Recent studies indicate a clinically significant increase in VTE risk associated with immune checkpoint inhibitor (ICI) therapy. A large retrospective cohort reported a cumulative incidence of approximately 10% at 6 months and up to 15% at 12 months, independent of cancer type or stage [34]. Additionally, a systematic review and meta-analysis found a pooled VTE incidence of 3.7 per 100 person-years, with the highest risk observed among patients receiving combination ICI therapies [35]. ICIs are also reported to increase the levels of proinflammatory cytokines, such as IL-6 and TNF-a, promoting the expression of tissue factor on endothelial cells and monocytes—initiating the extrinsic coagulation pathway [36]. An increased risk associated with immune check point inhibitors in some studies, especially when treatment exceeds 6 months, suggests that an extended overall survival and exposure to treatment in these patients may also be responsible for these differences [1,37].

Hospitalizations for an acute medical illness and the perioperative period are also linked to an increased risk for VTE in patients with cancer. Duration of inpatient stay and type of surgery (with general surgery and gynecological surgery posing the highest risk) also determine the risk for thrombosis and should be considered along with patient characteristics and cancer-related factors. In the prospective RISTOS study of more than 2300 patients undergoing cancer surgery, clinically overt VTE was assessed up to 30 ± 5 days postoperatively, with nearly 40% of events occurring later than 21 days after surgery; notably, VTE emerged as the leading cause of death within 30 days of surgery [14,38].

Altogether, the constellation of patient characteristics, cancer subtypes, treatment exposures, and biomarkers underscores the multifactorial nature of CA-VTE. These factors provide a foundation for understanding why thrombotic risk varies so widely across oncology populations, but they remain difficult to interpret in isolation. To address this challenge, researchers have sought to integrate these variables into formal risk prediction models that can distill complex clinical and biological information into tools usable at the bedside.

## 3. Risk Prediction of Cancer-Associated VTE

With advancements in diagnostics and delivery of care, objective measures like lab parameters can be a useful addition to risk prediction models [39]. Some clinical laboratory parameters, such as hemoglobin, white blood count and platelet count along with biomarkers such as D-Dimer and soluble P-selectin, have already been added as variables to risk assessment models to improve their predictive potential [40,41,42,43]. It is important to emphasize that D-dimer has high sensitivity but poor specificity and may be elevated in inflammatory and non-malignant conditions. The TiC-Onco score, which has been proposed as a risk-assessment model, utilizes novel genetic polymorphisms (e.g., SNPs in F5, F13, SERPINA10, and ABO loci) as a predictive variable [44]. Numerous biomarkers have been studied in primary and recurrent VTE in cancer patients but have not been included in risk assessment models [45]. The extent to which each of these variables impact predictive power remains largely unknown. Limited human studies, issues related to commercial availability and reliability of biomarkers pose a great challenge to their utility in risk prediction [46,47,48,49,50,51,52,53,54,55]. The invasive strategies required for some other biomarkers, such as brain tumor podoplanin, restrict their potential use in clinical practice [56]. Newer techniques like next-generation proteomic platforms and data-independent acquisition mass spectrometry can provide better assessment and screening of novel biomarkers [57]. However, these techniques are far from being widely available. Ultimately, an ideal biomarker must balance accuracy and predictive value with feasibility and cost-effectiveness to be usable in oncology practice. Considerable progress is still required to equip clinicians with reliable biomarker-based tools for CA-VTE risk prediction.

### Biomarker Assessments

Some biomarkers have been extensively investigated as predictive variables for the risk of CA-VTE. Leukocytosis, in particular neutrophilia and monocytosis, is associated with an increased risk of VTE in cancer patients. The ANC Study Group Registry and the Registro Informatizado de la Enfermedad Trombo-Embólica (RIETE) demonstrated a 1.6–2-fold increased risk of recurrent VTE in patients with elevated white blood cell count at baseline [40,58]. Similarly, thrombocytosis > 337,000/mm^3^ and hemoglobin < 10 g/dL were found to be associated with an increased VTE risk [15,59]. Soluble P-selectin, a thrombogenic cell adhesion molecule, has been found to be independently associated with a risk of cancer thrombosis [60]. In a study by Ay et al., the presence of both D-dimer and prothrombin fragment 1 + 2 (F1 + 2) led to a higher cumulative incidence of VTE [61]. While the Vienna Cancer and Thrombosis Study demonstrated that D-dimer and prothrombin fragment 1 + 2 levels were predictive of VTE in cancer patients, these findings were derived from a single-cohort analysis without external validation. F1 + 2 is not widely available in routine laboratories and its incorporation into clinical risk models limits real-world applicability.

Although these findings underscore the biologic plausibility of hematologic and molecular markers as predictors of CA-VTE, most studies remain limited by single-cohort designs, lack of external validation, and heterogeneity in measurement. Without rigorous validation across independent populations, their clinical utility remains uncertain. The limitations of conventional biomarkers have prompted the exploration of newer technologies such as next-generation proteomic platforms and advanced mass spectrometry. These approaches offer the potential to systematically identify and validate novel biomarkers, though their current use remains largely confined to research settings.

Ultimately, the promise of biomarkers lies in their integration into comprehensive risk models that combine clinical, therapeutic, and molecular variables. Realizing this potential will likely require parallel advances in computational approaches, including artificial intelligence and machine learning, to manage the complexity of multimodal data and translate it into clinically actionable risk prediction.

## 4. Current Risk Prediction Models

To translate this understanding of risk into practice, several predictive models have been developed over the past two decades. These models aim to formalize risk stratification by combining patient-, tumor-, and treatment-related factors into scoring systems that can guide prophylaxis and management decisions. Beginning with the widely adopted Khorana score, and extending to subsequent iterations such as PROTECHT, CONKO, Vienna-CATS, and disease-specific scores, these tools represent important steps toward the systematic prediction of CA-VTE. Examining their development, validation, and limitations provides key insights into how the field has evolved and where opportunities remain for refinement.

### 4.1. Khorana Score

Developed by Khorana and colleagues, the Khorana Score is a clinical model that estimates the risk of VTE in cancer patients initiating chemotherapy [40]. It was constructed using data from a large prospective study with derivation (*n* = 2701) and validation cohorts (n = 1365) [40]. The demographics within the study included ambulatory adult patients with a variety of cancer types who had not yet received chemotherapy, but anticipated at least four cycles of therapy [39]. The parameters for the predictive score included tumor site risk category (very high risk: pancreatic and gastric cancer; high risk: lung, lymphoma, gynecologic, bladder, testicular), laboratory parameters (hemoglobin < 10 g/dL; leukocyte count 11 G/L; platelet count ≥ 350 × 10^9^/L), and body mass index (≥35 kg/m^2^) [39]. Amongst the total study population of 4066 patients, 2.2% percent of the derivation cohort experienced VTE across a median follow-up of 73 days [39,40]. Across both derivative and validation cohorts, the rates of VTE were 0.8% and 0.3% in low-risk (score 0), 1.8% and 2% in intermediate-risk (score 1–2), and 7.1% and 6.7% in high-risk (score > 3) [40]. In both cohorts, the C-statistic was 0.7 [40].

### 4.2. PROTECHT, CONKO and Vienna Score

While the Khorana score is a leading contemporary model in assessing the risk of VTE, there are some limitations of this risk assessment model (discussed in detail below). As a result, multiple other risk assessment models have been developed to increase the ability to predict VTE in cancer patients. One prediction model is the PROTECHT score, designed by implementing modifications to the Khorana Score to increase its sensitivity. In the PROTECHT score, therapy criteria were enhanced to include platinum-based and/or gemcitabine chemotherapy (each adding + 1 point), in addition to the predictive variable criteria of the Khorana Score [42]. The PROTECHT risk model was a post hoc analysis of data from the placebo cohort of a prospective interventional study of 378 patients. The analysis demonstrated that 4.0% of the patients developed VTE, which included 8.1% in the high-risk population (≥3 points), and 2.0% in the low-risk population. Overall, 67% of the high-risk population accounted for all the VTE occurring in the patients. The CONKO score is another risk model that includes data from 312 consecutively recruited patients of the CONKO-004 trial [43]. To improve predictive utility, the CONKO model replaced BMI with the patient’s WHO performance status, reflecting functional capacity as a relevant risk factor (+1 point for a WHO performance status of ≥2). In addition, the Vienna modification of the Khorana score included D-dimer and soluble P-selectin, including data from 819 patients with relapsed cancer in a prospective observational cohort study [62]. The modifications proposed in the PROTECHT, CONKO and Vienna models were externally validated via a prospective cohort study. To evaluate the discriminatory performance of the scores, the time-dependent concordance index (c-index) was calculated while accounting for death as a competing risk. The results indicated a C-index of 0.50 (95% CI: 0.42–0.57) for the Khorana score, 0.54 (0.45–0.63) for the PROTECHT score, 0.50 (0.44–0.57) for the CONKO score, and 0.57 (0.48–0.66) for the Vienna modification of the Khorana score [63].

Thus, this external validation indicated that these models did not achieve a clinically important improvement in predictive performance. This highlights the ongoing need for more robust and individualized risk assessment tools for VTE in oncology.

### 4.3. ONKOTEV Score

The ONKOTEV score was developed based on four risk factors: presence of metastasis, compression of vascular structures, prior history of VTE, and a Khorana score greater than two [64]. Within a retrospective cohort of 165 patients with pancreatic cancer, an ONKOTEV score greater than two (43.6% of patients) had an incidence of VTE of 58% [65]. Martín et al. proposed the Tic-Onco risk score, calculated during patients’ initial cancer diagnosis to evaluate clinical and genetic risk factors for thrombosis. With data included from 391 outpatients with a recent cancer diagnosis and candidacy for chemotherapy, the Tic-Onco model employs genetic risk scoring via identifying single-nucleotide polymorphism (SNP) risk alleles. The following clinical variables are also included the following: primary tumor site, tumor node metastasis stage, body mass index (BMI), use of tobacco, age, sex, family (first degree) history of VTE, the presence of diabetes, hypertension, and high blood cholesterol level, the Khorana score, previous surgery, platelet count, number of leukocytes, and immobilization. An internal validation recorded 71 VTE events with improved predictive capability and sensitivity in comparison to the Khorana Score (AUC 0.73 vs. 0.58, sensitivity 49 vs. 22%) [44]. The study found time-dependent AUCs at 3, 6, and 12 months of 0.70 (95% CI, 0.621–0.787), 0.73 (95% CI, 0.656–0.791), and 0.72 (95% CI, 0.652–0.773), respectively. It should be noted that the incorporation of SNP risk alleles in routine oncology practice is currently challenging.

### 4.4. COMPASS-CAT

Developed through a multicenter prospective study of 1023 patients, the COMPASS-CAT score is a clinical tool designed to evaluate VTE risk in individuals with cancers such as breast (61%), colorectum (17%), lung (13%), and ovarian malignancies (7%). The model incorporates variables including type and timing of therapy (e.g., antihormonal or anthracycline use), cancer stage, comorbid cardiovascular conditions, recent hospitalizations, platelet count, presence of a central venous catheter, and prior thromboembolic events [66]. Patients categorized as a low/intermediate risk had a 6-month VTE rate of 1.7% in comparison to 13.3% within high-risk populations. Within the derivation cohort, the study achieved a C-statistic of 0.85, in comparison to an external validation study (n = 3814), which had a C-statistic of 0.62 [66,67].

### 4.5. Ottawa Score

The Ottawa score was developed as another risk model to stratify patients according to their risk of recurrent VTE. Louzada et al. conducted a retrospective cohort study (n = 543) with four independent variables (sex, primary tumor site, stage, and prior VTE), demonstrating a 98.1% negative predictive value, 48.1% low-risk excluded proportion and a negative likelihood ratio of 0.155 [68]. However, the Ottawa score has shown poor results in recent external validation studies and thus its use is not currently recommended to assess the risk of VTE recurrence in cancer patients [69,70].

### 4.6. Myeloma-Specific Models

The SAVED-score risk assessment model was developed as a disease-specific risk-stratification model for patients with multiple myeloma (MM). The pathophysiology and the treatment for MM (immunomodulatory drugs like lenalidomide or thalidomide) both lead to an increase in the risk of VTE [71]. A retrospective cohort study was conducted, where patients were included from the SEER-Medicare database (n = 2397). In the derivation cohort, five clinical variables were selected in the inclusion criteria, where patients had a 6-month VTE risk of 12%, compared to 7% in the low-risk group [72]. The SAVED-score model had a C-statistic of 0.61, and was confirmed in an independent external validation study (n = 501, C statistic = 0.74) [72,73]. Similarly, the IMPEDE VTE score was developed for MM patients undergoing chemotherapy treatment. Through a retrospective study (n = 4466), nine clinical variables were selected in the inclusion criteria and patients were stratified in risk groups where the incidence of cumulative 6-month VTE was 15.2% in the highest risk group (10.1% of patients), in comparison to 8.3% in the intermediate-, and 3.3% in the low-risk category [74]. The model had a C-statistic of 0.66 in the derivation cohort, and 0.68 in the external validation [74,75].

See Table 1 for an overview of established risk assessment models for cancer-associated thrombosis, showcasing specific cancer/tumor subtypes, risk factors, and their associated points, risk categories, and overall performance.

## 5. Gaps and Pitfalls in Existing Models

CA-VTE causes a significant impact on survival, quality of life and healthcare costs [4,82]. Its diagnosis increases healthcare utilization, requiring more frequent clinical assessments. Additionally, the treatment of CA-VTE carries the risk of increased bleeding.

There are multiple risk prediction models for CA-VTE [39,40,63,83,84]. While the Khorana score is currently the most established risk model for predicting CA-VTE, it has some limitations [85,86]. The most important limitation is that it does not account for newer cancer therapies, several of which are prothrombotic. With improved cancer care, the sensitivity of the score has also been questioned. In a Danish cohort of ~40,000 cancer patients, the VTE risk was much lower in patients with a Khorana score ≥ 2 compared to previous reports [14].

The decision to initiate thromboprophylaxis in cancer patients is further complicated by the limited discriminatory ability of existing risk models and the competing risk of bleeding. Anticoagulation trials in ambulatory cancer patients have consistently demonstrated that while prophylaxis lowers VTE risk, clinicians must manage the cost of bleeding complications. The AVERT study demonstrated that apixaban lowered the incidence of VTE in high-risk ambulatory cancer patients. However, this benefit was accompanied by an increased risk of major bleeding events, which occurred in approximately 2% of patients receiving apixaban versus 1% in the placebo group, while clinically relevant non-major bleeding occurred in 7.3% of patients [87]. Similarly, in the CASSINI trial, rivaroxaban lowered VTE incidence but was associated with major bleeding in 2.0% of patients, in comparison to 1.0% in the placebo cohort [88]. Bleeding risk is particularly pronounced in patients with gastrointestinal and genitourinary cancers, where mucosal tumor involvement further predisposes the patient to hemorrhage [89,90].

Alongside bleeding risk, limitations in predictive accuracy complicate thromboprophylaxis decisions. A 2019 meta-analysis of 55 cohorts (N = 34,555) demonstrated that only 11% of high-risk patients (score ≥ 3) developed VTE within 6 months, while approximately 76% of VTE events occurred in low- or intermediate-risk groups—underscoring the Khorana score’s limited sensitivity [85]. However, when a lower threshold of ≥2 was used, the proportion of VTEs captured increased to 55%, but nearly half of the patients were classified as high-risk, diminishing specificity. The dual challenge of imperfect risk prediction and clinically meaningful bleeding risk underscores the urgent need for integrated models that can simultaneously account for thrombotic and bleeding risk, thereby guiding safer and more individualized prophylaxis strategies in cancer care.

This complexity is heighted by several factors, such as tumor location, invasion of vascular structures, chemotherapy-induced thrombocytopenia, and frequent invasive procedures—all of which elevate the risk of bleeding independent of coagulation [89,90]. Thus, clinicians are forced to balance the morbidity and mortality of VTE against potentially life-threatening bleeding complications, making thromboprophylaxis decisions highly nuanced and complex. These challenges underscore the need for both improved models, that accurately predict thrombotic and bleeding risks, and safer anticoagulants, in order to provide effective and safe anticoagulation strategies in cancer care.

Race and ethnicity are also associated with CA-VTE with African Americans having higher incidence of CA-VTE in most cancer subtypes [16,17]. This could be related to racial disparities in access to health care, leading to the presence of an advanced stage and preexisting comorbidities at the time of cancer diagnosis. However, African Americans have been underrepresented in patient cohorts used for risk model development, making these tools unreliable in certain patient populations [17]. This suggests that addition of race/ethnicity as a variable in future risk prediction models could provide more optimal risk stratification.

Another potential variable for predicting risk for CA-VTE is the class of systemic therapy that a cancer patient is exposed to. The PROTECHT and COMPASS-CAT are the only risk prediction models that have addressed the role of prothrombotic chemotherapeutic and hormonal agents (e.g., platinums, gemcitabine, anthracycline and antihormonal therapy) [42,66]. Some additional systemic therapies such as immune checkpoint inhibitors, anti-angiogenic agents and tyrosine kinase inhibitors have been associated with a prothrombotic risk. Incorporation of these in future risk prediction models is imperative for improved accuracy.

Efforts to improve CA-VTE prediction should emphasize refinement of strategies that incorporate validated biomarkers, systemic therapy class, and sociodemographic determinants to optimize discriminatory performance. The application of advanced computational techniques, including artificial intelligence and machine learning, offers the potential to create dynamic models responsive to changes in oncology practice and patient heterogeneity. Such frameworks may improve identification of high-risk individuals and support safer, more individualized prophylaxis strategies

## 6. Recommendations on Prophylaxis

Guidelines on thrombophylaxis in various settings provide a framework for clinicians when managing cancer patients and the risk of VTE [91,92,93,94,95,96]. Stronger data are needed to add strength to the level of evidence for each recommendation. However, as suggested by all guidelines, the decision to proceed with primary VTE prophylaxis in cancer patients needs to be based upon a patient–physician discussion on the risk–benefit profile of anticoagulation therapy in an individualized setting. Continual assessment of risk is essential, as it may change with improving or worsening cancer status. Incorporation of genetic polymorphisms and proteomic profiling into risk prediction models can help increase the sensitivity of risk models, providing improved risk assessment. Novel anticoagulants with a reduced bleeding risk may also help simplify decision-making in this regard. We recognize that genetic polymorphisms and proteomic profiling remain investigational at this time, and clinicians should rely on validated clinical risk scores.

In Table 2 the authors present risk categories and proposed thromboprophylaxis guidelines for CA-VTE.

## 7. Prevention and Management of Cancer-Associated Thrombosis

### 7.1. Primary Prevention in Ambulatory Patients

While the routine use of primary thrombophylaxis is not indicated in all ambulatory cancer patients, multiple international clinical practice guidelines now recommend considering the use of thrombophylaxis in high-risk outpatients (Khorana score ≥ 2) undergoing treatment on an individualized basis [91,92,95,96,97,98]. However, the use of prophylactic anticoagulation comes with the risk of increased bleeding. Treatment with low molecular weight heparin (nadroparin or semuloparin) has previously been shown to reduce VTE rates in ambulatory cancer patients on systemic chemotherapy [99,100]. The leading trials on anticoagulation in cancer patients, namely PHACS, CASSINI and AVERT, have used the Khorana score to risk stratify the patients.

In the PHACS, study patients diagnosed with cancer were randomized and recruited with the criteria of a Khorana score ≥ 3 to prophylactic anticoagulation with a Low Molecular Weight Heparin (LMWH) or observation for 12 weeks. The results indicated a non-significant 9% absolute risk reduction in VTE (hazard ratio: 0.64 (95% confidence interval, CI: 0.22–1.72)) and a significant increase in clinically relevant bleeding events (HR: 7.02; 95% CI: 1.24–131.6) within the LMWH group [101].

The CASSINI and AVERT trials were randomized placebo-controlled clinical trials, evaluating the safety and efficacy for ambulatory patients with cancer. These patients with a Khorana score of two or more received primary prophylaxis with a direct oral anticoagulant (DOAC). Patients treated with rivaroxaban demonstrated a 6-month decrease in VTE risk (2.6% vs. 6.4%, hazard ratio (HR: 0.40; 95% CI: 0.20–0.80)), together with a non-significant increase in major bleeding events (2.0% vs. 1.0%, HR: 1.96; 0.59–6.49) [88]. Similarly, the AVERT trial showcased that treatment with apixaban led to a decrease in VTE rates (4.2% vs. 10.2%, HR: 0.41; 95%CI: 0.26–0.65), but caused an increase in major bleeding complications (2.1% vs. 1.1%, HR: 1.89; 95% CI: 0.39–9.24) [87].

In a recent meta-analysis of randomized controlled trials by Myat et al. on outpatient thrombophylaxis in patients with cancer with Khorana score ≥ 2 receiving chemotherapy, the VTE incidence was down to 5.20% and as much as 1.54% in patients receiving DOACs and LMWH respectively (RR of 0.56 vs. 0.22). This apparent discrepancy can be explained by several important methodological- and population-level differences. First, the pooled analysis focused on patients with a Khorana Score (KS) ≥ 2, whereas some landmark trials enriched higher-risk subsets (e.g., KS ≥ 3) or a higher thrombogenic cancer type (such as pancreatic cancer), resulting in different baseline thrombotic risk profiles. Second, heterogeneity across trials and variations in endpoint definitions (symptomatic vs. composite symptomatic plus incidental VTE) and follow-up duration further contribute to differences in reported VTE incidence. Third, the LMWH group included intermediate and therapeutic doses which may account for the differences in relative benefit. Finally, pooled analyses inherently average across diverse study designs and eras, whereas individual randomized trials reflect more internally consistent populations. Taken together, these factors likely account for the differing relative estimates between LMWH and DOACs [102].

Most international guidelines on prevention of CA-VTE suggest the preferential use of DOACS compared to LMWH in the absence of any contraindications (e.g., gastrointestinal or genitourinary malignancy) or drug–drug interactions. The International Initiative on Thrombosis and Cancer (ITAC) and the Spanish Society of Medical Oncology (SEOM) also incorporated certain high-risk cancer types (advanced and metastatic pancreatic cancer) in their recommendations [93,96,97]. Similarly, recommendations are in place for patients with MM regarding the use of aspirin and LMWH based on their risk stratification (low- or high-risk, respectively) [93,95,97,98]. While the SEOM guidelines also include non-small cell lung cancer with ROS-1 and ALK rearrangement in the high-risk category where thrombophylaxis should be considered, the more recent ITAC guidelines do not recommend its use outside of a clinical trial due to increased risk of bleeding and no difference in survival [93,96].

### 7.2. Primary Prevention in Hospitalized Patients

While the risk of VTE is significantly increased in hospitalized cancer patients, there is limited consensus regarding primary prophylaxis [38]. The results from the ARTEMIS, PREVENT and Enoxaparin trials did not demonstrate a significant benefit of primary thrombophylaxis in this patient cohort. Small sample size is the biggest limitation for these trials, therefore reducing generalizability [103,104,105]. Multiple international guidelines have conflicting guidelines in regard to primary prevention in these patients. The American Society of Clinical Oncology (ASCO), International Society of Hemostasis and Thrombosis (ISTH) and SEOM all recommend anticoagulation prophylaxis in cancer patients who have a concurrent acute medical illness without any contraindications [98,106,107]. ASCO recommends against thrombophylaxis in patients who are admitted for stem cell transplants or chemotherapy due to limited benefit in the face of increased bleeding rates in these patients. ISTH recommends individualized decision-making for use of anticoagulation in patients with thrombocytopenia. ITAC and the National Comprehensive Cancer Network (NCCN) recommend anticoagulation prophylaxis in all hospitalized patients regardless of coexisting medical illness [93,95,97]. DOACs are usually not recommended in hospitalized patients; LMWH is the usual preferred agent.

### 7.3. Primary Prevention in Surgical Patients

The risk of venous thromboembolism in the postoperative period continues to remain high for several weeks after a major surgery in a cancer patient [108]. In the ENOXACAN study, for the group of cancer patients undergoing abdominal or pelvic surgeries, enoxaparin was established as having superior efficacy with similar bleeding complication when compared to unfractionated heparin [109]. The international guidelines on cancer thrombosis recommend thrombophylaxis with LMWH or UFH to be initiated preoperatively and to continue for 7–10 days after the surgery [93,95,97,98,106]. Extended-duration thromboprophylaxis for up to 4 weeks is recommended in high-risk patients undergoing abdominal or pelvic cancer surgery (high-risk is categorized as those patients with restricted mobility, obesity, previous VTE, or additional risk factors, anesthesia time > 2 h, gastrointestinal malignancies, previous VTE, advanced-stage disease, bed rest of ≥4 days, or age > 60 years) [95,106]. While LMWH was initially recommended for extended prophylaxis, ASCO recently updated their guidelines, adding potential use of rivaroxaban or apixaban in this setting as a weak recommendation [94]. This recommendation is based on two recent RCTs reporting safe and efficacious use of these DOACs in cancer patients undergoing major surgeries [110,111]. The use of mechanical prophylaxis or inferior vena cava filter is not recommended in the absence of anticoagulation contraindication.

## 8. Treatment of Cancer-Associated Thrombosis

Patients with CA-VTE are heterogenous and the choice of anticoagulant is dependent upon multiple factors, such as patient characteristics and preferences, cancer subtype and drug–drug interactions. Multiple RCTs involving head-to-head comparisons of different anticoagulants have allowed guideline recommendations in patients with CA-VTE. We summarize some treatment options below.

### 8.1. Low-Molecular-Weight Heparin (LMWH)

The CLOT trial was the landmark study that established LMWH as the preferred agent for VTE treatment in cancer patients [112]. It demonstrated improved VTE recurrence rates with dalteparin when compared to warfarin (6 months; 17 vs. 9%) and similar safety and mortality outcomes. Alongside the CLOT trial, the CATCH and RIETECAT supported LMWH as an effective standard therapy for cancer-associated thrombosis prior to the widespread adoption of DOACs [113,114]. While some other RCTs failed to consistently prove a superior efficacy of LMWH over oral vitamin K antagonist (VKA) agents, multiple systemic metanalysis studies published results in favor of LMWH [115,116,117,118,119]. The challenges associated with VKA monitoring and the data regarding the safety and efficacy of LMWH led to its adaptation as standard first-line therapy. Currently, all international guidelines recommend the use of LMWH as one of the possible strategies when dealing with treatment of CA-VTE [78,79,93,97,98,106].

### 8.2. Direct Oral Anticoagulants

With the advent of DOACs and their favorable side effect profile, reduced need for monitoring and ease of administration makes them a preferred option among clinicians and patients alike. However, caution is advised in certain situations as detailed below.

In 2018, two RCTs, SELECT-D [80] and Hokusai [81], studied the use of DOACs in CA-VTE. In the SELECT-D trial, the treatment with rivaroxaban reduced the occurrence of VTE at 6 months compared to dalteparin (6% vs. 11%, HR 0.43). However, this benefit was offset by a higher frequency of non-major bleeding events (13% vs. 4%), emphasizing the importance of individualized risk–benefit assessment in anticoagulation choice. Among patients with gastrointestinal cancers, there was a non-significant increase in major bleeding episodes which prompted the exclusion of these patients from the trial. In the Hokusa–VTE cancer study comparing edoxaban to dalteparin, the rate of recurrent VTE was numerically lower with edoxaban (7.9% vs. 11.3%, HR 0.71; 95% CI 0.48–1.06), but this was offset by a significantly higher rate of major bleeding (6.9% vs. 4.0%; HR 1.77; 95% CI 1.03–3.04), particularly in patients with gastrointestinal cancer (12.7% vs. 3.6%). In the meta-analysis by Li et al., DOACs overall were associated with a non-significant reduction in recurrent VTE (RR 0.65; 95% CI 0.42–1.01), but a significantly higher incidence of major bleeding compared with LMWH (RR 1.74; 95% CI 1.05–2.88) [120]. A real-world study under the OSCAR-US program, published in 2023, reported a decreased risk of VTE at 3 and 6 months with no difference in bleeding-related hospitalizations or mortality when rivaroxaban and LMWH were compared [121]. Of note, patients with cancers with an established high risk of bleeding on DOACs were excluded in this study. CASTA-DIVA, another open-label, randomized, non-inferiority trial, compared rivaroxaban with dalteparin for the treatment of cancer-associated thrombosis; the study was terminated early because of slow accrual. Recurrent VTE occurred in 6.4% of patients receiving rivaroxaban and 10.1% receiving dalteparin, while major bleeding occurred in 1.4% and 3.7%, respectively. The trial was underpowered to demonstrate non-inferiority [122].

Three recent head-to-head trials of apixaban versus LMWH have established apixaban as a first-line agent in most cases of CA-VTE unless a contraindication is present [123,124,125]. The CARAVAGGIO trial, an open-label non-inferiority study comparing oral apixaban and subcutaneous dalteparin, had two primary efficacy and safety outcomes: recurrent VTE and major bleeding. The Caravaggio trial reported that apixaban was non-inferior to dalteparin in preventing recurrent VTE, with recurrence rates of 5.6% in the apixaban group versus 7.9% with dalteparin (HR 0.63, 95% CI 0.37–1.07), demonstrating non-inferiority [126]. Major bleeding rates were similar between the two groups, occurring in 3.8% of apixaban patients and 4.0% of dalteparin patients (HR 0.82, 95% CI 0.40–1.69) [126]. Clinically relevant non-major bleeding was reported in 9.0% with apixaban versus 6.0% with dalteparin (HR 1.42, 95% CI 0.88–2.30). In a subgroup analysis of the CARVAGGIO trial [126], patients with GI and GU cancers were at the highest risk of recurrent VTE and major bleeding. Notably, however, there was no difference in these outcomes between the two anticoagulant arms across patients with other primary cancer sites.

ADAM-VTE trial also compared apixaban to dalteparin for CA-VTE treatment [125]. Major bleeding occurred in none of the 145 patients in the apixaban group and 1.4% of 142 patients in the dalteparin group. Rates of recurrent VTE were significantly lower in the apixaban arm compared with the dalteparin arm (0.7% vs. 6.3%, HR: 0.099; 95% CI 0.013 to 0.780). The frequency of CRNMB was 6% in both groups. The bleeding rate for the ADAM-VTE trail was lower than other trials comparing apixaban and dalteparin. This could be possible due to a lower proportion of GI and GU cancers in the ADAM-VTE cohort. Another trial compared apixaban and enoxaparin and did not report any differences in recurrent VTE and major bleeding events between the two arms [124].

Based on these studies showing non-inferiority of DOACs to LMWH in terms of VTE recurrence, the use of DOACs have been incorporated in almost all guidelines, with NCCN and ISTH preferentially recommending DOACs over LMWH unless there is a high risk of bleeding, drug–drug interactions or other contraindications [79,95,127,128]. LMWH is the preferred class in GI or GU cancers. Overall, guidelines recommend anticoagulation for as long as cancer is active or treatment is ongoing with a minimal duration of 3–6 months [78,79,93,95,97,98,106]. ASCO and NCCN also recommend treating incidental VTE in the same manner as a symptomatic VTE [95,98]. IVC filters are usually not recommended in these guidelines unless there is a long-term contraindication to anticoagulation or thrombus progression despite full-dose anticoagulation. Recurrent breakthrough VTEs require that a clinician does a thorough assessment of potential contributing reasons, such as medication adherence, drug–drug interactions, inappropriate dose reductions or etiologies leading to poor oral drug absorption. If any such issues are not identified, switching from prophylactic to therapeutic dosing between different classes of anticoagulants is acceptable [93,95,97].

**Figure 3:**
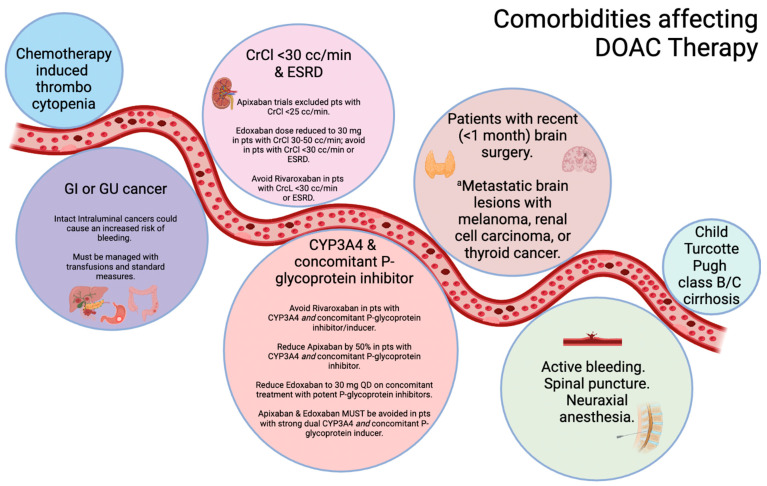
Specific patient populations requiring cautious use of DOAC therapy for cancer-associated venous thromboembolism. Created in BioRender. Khan, S. (2026) https://BioRender.com/2uybnqs (accessed on 22 January 2026). ^a^ There is growing literature regarding the better safety and efficacy profile of DOACs compared to LMWH in central nervous system cancers. Abbreviations: CrCl: creatine clearance; CC: cubic centimeter; GI: gastrointestinal; GU: genitourinary; Min: minute; Pts: patients.

The oral administration of DOACs makes it a convenient option with better patient adherence. However, some special populations with CA-VTE require the judicious use anticoagulation, especially DOACs (Figure 3). Overall, the selection of anticoagulant needs to be individualized, considering both patient- and cancer-related risk factors, and associated comorbidities, drug–drug interactions, patient preferences and the cost of the drug.

### 8.3. Selective Parenteral Indirect Factor Xa Inhibitor

Fondaparinux is a synthetic pentasaccharide that functions as a selective factor Xa inhibitor through the antithrombin-mediated mechanisms, potentiating factor Xa neutralization by approximately 300-fold [129]. Unlike heparin and other LMWH, fondaparinux does not bind to platelet factor 4 (PF4), conferring negligible risk of heparin-induced thrombocytopenia (HIT) [130]. The agent exhibits predictable pharmacokinetics with complete subcutaneous bioavailability (100%), consisting of a half-life of 17–21 h permitting once-daily dosing, and renal excretion without metabolism. These properties eliminate the need for routine laboratory monitoring in most patients [130,131].

In patients with lower extremity superficial vein thrombosis (SVT), at least 5 cm in length located more than 3 cm from the saphenofemoral junction, and the CALISTO trial demonstrated that having fondaparinux (2.5 mg) subcutaneously once daily for 45 days significantly reduced the composite outcome of mortality, symptomatic venous thromboembolism (VTE), and SVT extension or recurrence compared with placebo (0.9% vs. 5.9%; relative risk 0.15, 95% CI 0.08–0.26) [132,133]. Deep vein thrombosis (DVT) or pulmonary embolism (PE) occurred in 0.2% of fondaparinux-treated patients versus 1.3% of placebo recipients, with comparable major bleeding rates (0.1% each group). Professional societies recommend fondaparinux (2.5 mg) daily for 45 days as first-line therapy for lower extremity SVT ≥5 cm in length and >3 cm from the saphenofemoral junction [132,133]. For lower extremity SVT in cancer patients, NCCN recommends consideration of prophylactic dose anticoagulation for at least 6 weeks if SVT ≥ 5 cm in length and above the knee [35,134]. Therapeutic dose anticoagulation for at least 3 months is recommended for SVT within 3 cm of the saphenofemoral junction [134]. Symptomatic management may be sufficient for catheter-related upper extremity SVT if the catheter is removed with a low threshold to consider anticoagulation, with evidence of progression or clot in close proximity to the deep venous system [134].

Fondaparinux is recognized in the ESMO Clinical Practice Guidelines as an acceptable parenteral anticoagulant for the initial management of cancer-associated venous thromboembolism, particularly during the first 5–10 days or longer when rapid and reliable anticoagulation is required. Its inclusion alongside low-molecular-weight and unfractionated heparins highlights its clinical utility in selected patients, especially those with contraindications to heparins such as heparin-induced thrombocytopenia, intolerance to heparin products, or situations in which oral anticoagulants are not appropriate [78]. Fondaparinux offers advantages in heparin-induced thrombocytopenia, with propensity-matched studies demonstrating comparable effectiveness and safety to approved non-heparin anticoagulants [130,135]. Despite guideline inclusion, fondaparinux remains underrecognized among oncologists, reflecting its lower grade of recommendation compared with LMWH (1A) and direct oral anticoagulants (1A), limited cancer-specific trial data, and absence from simplified treatment algorithms. Explicit inclusion in treatment pathways would enhance appropriate utilization in suitable clinical scenarios [93].

## 9. Duration of Anticoagulation and Potential for Low-Dose Regimen Use

Typically, the duration of anticoagulation for VTE treatment ranges from 4 to 12 weeks and beyond 12 weeks in certain situations. The duration can differ depending on indication, for example, treatment versus primary or secondary prophylaxis. Current guidelines suggest extending anticoagulant treatment if the patient has active cancer but evidence for anticoagulation beyond 6 months in cancer populations is limited to a few prospective studies [127,136,137]. Extended treatment with low molecular weight heparin has been evaluated in several prospective studies. In the DALTECAN trial, patients received dalteparin for up to 12 months, and the majority of thrombotic recurrences were observed within the initial 6-month period, while bleeding rates remained relatively constant beyond that time [127]. Comparable results were reported in the TiCAT study, which examined tinzaparin therapy and demonstrated persistently low rates of both recurrent VTE and major bleeding during the 6–12 month treatment interval [136]. Together, these studies support the safety and effectiveness of prolonged LMWH therapy in appropriately selected cancer patients. In contrast, the Hokusai VTE Cancer trial, which followed patients for 12 months, found a lower rate of VTE recurrence with edoxaban compared with dalteparin (7.9% vs. 11.3%), but this benefit was offset by significantly higher rates of major bleeding, particularly in GI cancers [137]. Collectively, these findings suggest that extended therapy is feasible and generally safe with LMWH, whereas DOAC use beyond 6 months may require caution in patients with high bleeding risk malignancies.

Within the general population, secondary prophylaxis with reduced-dose apixaban (2.5 mg twice daily) and rivaroxaban (10 mg daily) has been demonstrated to be protective against recurrent VTE without significantly increasing the rate of major bleeding—showcasing efficacy and safety [138,139]. Current guidelines on CA-VTE thus far continue to recommend a full-dose anticoagulation regimen for secondary prophylaxis beyond 3–6 months of primary treatment.

In an effort to explore the safety and efficacy of low-dose anticoagulation in cancer-associated VTE for secondary prophylaxis, a single arm interventional clinical trial was conducted in Norway where 298 patients were treated with full-dose apixaban (5 mg twice daily) for 6 months and 196 patients with active cancer after 6 months of treatment continued with apixaban 2.5 mg twice daily for another 30 months [140]. Through the 30-month treatment with low-dose apixaban, 14 patients (7.6%; 95% confidence interval (CI) 4.0–11.7%) experienced recurrent VTE, six patients (3.1%; 95% CI 1.1–6.5%) experienced major bleeding and 16 patients (8.1%, 95% CI: 4.7–12.8%) experienced clinically relevant non-major bleeding [140]. While there was a trend towards a small transient increase in VTE and a substantial decrease in major bleedings between 7 and 12 months, the incidence of recurrent VTE and major bleeding remained low with 2.5 mg of apixaban taken twice daily beyond 12 months. This study shows that a reduction in apixaban dosage to 2.5 mg is effective and safe.

The API-CAT phase 3 trial investigated whether a reduced-dose apixaban strategy could provide effective extended anticoagulation in patients with active cancer who had already completed at least 6 months of therapy. In this randomized, double-blind, multinational study conducted across 11 countries, 866 participants received apixaban 2.5 mg twice daily and 900 participants received the standard dose of 5 mg twice daily for an additional 12 months [141]. Recurrent VTE occurred in 18 patients in the reduced-dose group and 24 patients in the full-dose group, corresponding to cumulative incidences of 2.1% and 2.8%, respectively (adjusted subhazard ratio 0.76; 95% CI 0.41–1.41; *p* = 0.001 for non-inferiority). In the reduced-dose group, 102 patients (cumulative incidence, 12.1%) experienced clinically relevant bleeding, defined as major or clinically relevant non-major bleeding over 12 months (adjusted subhazard ratio, 0.75; 95% CI, 0.58 to 0.97; *p* = 0.03 for superiority), 24 patients (cumulative incidence, 2.9%) had major bleeding (adjusted subhazard ratio, 0.66; 95% CI, 0.40 to 1.10) and 84 patients (cumulative incidence, 10.0%) experienced clinically relevant non-major bleeding (adjusted subhazard ratio, 0.79; 95% CI, 0.59 to 1.05) [141]. These findings demonstrate that the reduced-dose regimen was non-inferior to the standard dose for prevention of VTE recurrence while showing a more favorable bleeding profile.

Secondary outcomes showed comparable rates of recurrent major VTE (2.0% vs. 2.4%) [subhazard ratio, 0.83; 95% CI, 0.44 to 1.57] and all-cause mortality (17.7% vs. 19.6%) between groups. The net clinical benefit, defined as the composite of recurrent symptomatic VTE, major bleeding, or all-cause death, was similar in both groups (19.9% vs. 22.1%; HR, 0.96; 95% CI, 0.87–1.07). Serious adverse events, such as GI, vascular, renal and nervous systems disorders, occurred in 39.8% of patients in the reduced-dose group and 43.8% in the full-dose group.

Overall, this study shows that a reduced-dose apixaban regimen was non-inferior to full-dose apixaban in the incidence of recurrent VTE at 12 months. In addition, clinically relevant bleeding occurred significantly less frequently in the reduced-dose group. However, this study was unable to collect racial/ethnic data, thus limiting the ability to perform risk stratification based on demographic subgroups.

In Table 3 the authors present anticoagulant regiments for CA-VTE cases according to the treatment protocol.

## 10. Special and Challenging Scenarios in CA-VTE

### 10.1. Thrombocytopenia

LMWH is the preferred therapy for cancer patients with thrombocytopenia [142]. Full-dose anticoagulation is considered relatively safe in patients with platelet count more than 50,000/µL. Dose modification to reduced-dose LMWH is recommended if platelet counts are 25,000–50,000/µL. For severe thrombocytopenia with platelet count < 25,000 and an acute CA-VTE event, platelet transfusion should be considered in order to provide safe anticoagulation [96]. Frequent platelet transfusion is not always clinically feasible.

### 10.2. Brain Cancers and Metastases

Anticoagulation appears to be well-tolerated in patients with gliomas and most metastatic cancers to the brain [143,144]. Cautious use of anticoagulation and dose modification is usually recommended in certain secondary cerebral nervous system cancers from melanoma and kidney cancer. There is growing literature regarding the safer use of DOAC compared to LMWH in this high-risk population [145].

### 10.3. Concurrent Antiplatelet Therapy

Concurrent antiplatelet therapy along with anticoagulation can significantly increase the risk of bleeding in a cancer patient. This regimen should be reserved for exceptional situations of a recent acute coronary syndrome event or vascular stent placement [96]. LMWH may be preferred over DOAC in such cases [142].

### 10.4. Catheter-Related Thrombosis

Thrombophylaxis for catheter-related thrombosis is not usually recommended in cancer patients. In patients who develop a catheter-related thrombus, treatment dose of anticoagulation is warranted for at least 3–6 months unless a contraindication exists. Beyond 3–6 months, if the catheter is maintained, anticoagulation should be extended (possibly at a lower dose) [146]. Catheter removal should be considered if it is no longer clinically required.

## 11. Clinical Trials in Progress

There are several ongoing multi-institutional clinical trials currently enrolling patients to investigate the prophylaxis and treatment strategies in CA-VTE.

The START trial (ClinicalTrials.gov NCT05255003) is a Phase 4 RCT, enrolling adult patients with acute CA-VTE (diagnosed within 14 days) and thrombocytopenia (platelet count < 50,000/µL) who will be randomized 1:1 to modified-dose LMWH or higher-dose LMWH with platelet transfusion support. The objective of this study is to evaluate the superiority of a modified dose LMWH strategy in reducing clinically relevant bleeding events compared to full-dose LMWH with platelet transfusion. The study is currently active and recruiting patients.

Abelacimab is a Factor XI inhibitor which has shown promising results for preventing VTE with a much better safety profile and the convenience of infrequent dosing [147]. NCT05171049 (ClinicalTrials.gov; ASTER trial), which compared abelacimab relative to apixaban in CA-VTE, and NCT05171075 (ClinicalTrials.gov; Magnolia trial), which evaluated the efficacy and safety of abelacimab and dalteparin to prevent recurrent VTE in patients with gastrointestinal or genitourinary cancers, have both been terminated prematurely following emerging concerns regarding inferior efficacy and potential harm with abelacimab compared with standard anticoagulation. The absence of a reversal agent for factor XI inhibitors and the implications for urgent surgery or trauma also make it particularly challenging to use in this high-risk population.

An early Phase 1 trial (ClinicalTrials.gov NCT02285738) is also ongoing to determine the efficacy of aspirin with and without simvastatin in solid tumor patients with high or intermediate risk for VTE, in reducing markers of platelet activation, inflammatory cytokines and measures of hemostatic activation. The trial has been completed, and participants are no longer being examined or treated.

## 12. Conclusions

Cancer-associated venous thromboembolism (CA-VTE) remains a major source of morbidity and mortality in oncology, driven by complex interactions between tumor biology patient-specific factors and cancer therapies. While risk models such as the Khorana score have advanced clinicians’ ability to identify high-risk patients, their limitations highlight the urgent need for more precise tools that incorporate biomarkers, genomics, and treatment-related factors. Direct oral anticoagulants (DOACs) have expanded the therapeutic landscape, but safety concerns, particularly in GI and GU cancers, underscore the importance of individualized decision-making. Current guidelines recommend anticoagulation beyond the initial treatment phase for patients with active cancer, though data on optimal duration and dosing remain limited. Emerging trials and novel agents, including factor XI inhibitors, are poised to refine prophylaxis and treatment strategies. Moving forward, addressing disparities in clinical trial representation, integrating real-world evidence, and applying precision medicine approaches will be essential to improving outcomes for patients with CA-VTE.

## Figures and Tables

**Table 1:** Overview of established risk assessment models for cancer-associated thrombosis, showcasing specific cancer/tumor subtypes, risk factors and their associated points, risk categories and overall performance [63,67,72,74,76,77]. Adapted from F. Moik et al., 2021 [39] with permission.

Risk-Score	Cancer/Tumor Subtypes	Risk Factors	Points	Risk Categories	C-Stat
Khorana Score	Breast 35%, Lung 21%, Lymph. 12%, CRC 11%, Gyn. 10%	Very high-risk tumor types High-risk tumor types Hb < 10 g/dL or ESA-use WBC > 11 G/L Platelets ≥ 350 G/L BMI > 35 kg/m^2^	2 1 1 1 1 1	0 (Low) 1–2 (Intermediate) ≥3 (High)	Derivative: 0.7 Int. Validation: 0.7 Ext. Validation: 0.61 [19,76], 0.52 [78]
Vienna CATS	Breast 17.1%, Lung 15.3%, Colorectal 13.7%, Prostate 13.7%, Brain 13.1%, Lymphoma 11.8%, Pancreas 5.7%, Stomach 4.4%, Kidney 2.9%, MM 2.2%	Very high-risk tumor types High-risk tumor types Hb < 10 g/dL or ESA-use WBC > 11 G/L Platelets ≥350 G/L BMI > 35 kg/m^2^ D-Dimer ≥ 1.44 μg/L sP-selectin ≥ 53.1 ng/L	2 1 1 1 1 1 1 1	0 (Low) 1–2 (Intermediate) ≥3 (High)	Derivative: n/a
PROTECHT	Gastrointestinal 38.8%, Lung 21%, Breast 14.4%, Ovary 12.3%, Pancreas 4.5%, Head and neck 4.5%, Other 4.5%	Very high-risk tumor types High-risk tumor types Hb < 10 g/dL or ESA-use WBC > 11 G/L Platelets ≥ 350 G/L BMI > 35 kg/m^2^ Gemcitabine therapy Platinum-based therapy	2 1 1 1 1 1 1 1	0–2 (Low-Intermediate) ≥3 (High)	Ext. Validation: 0.54 [78], 0.59 [78], 0.61 [79]
CONKO	Advanced pancreatic adenocarcinoma (100%)	Very high-risk tumor types High-risk tumor types Hb < 10 g/dL or ESA-use WBC > 11 G/L Platelets ≥ 350 G/L BMI > 35 kg/m^2^ ECOG performance status ≥2	2 1 1 1 1 1 1	0–2 (Low-Intermediate) ≥3 (High)	Ext. Validation: 0.50 [78], 0.53 [78], 0.60 [79]
ONKOTEV	Breast 36.6%, GI-/pancreatic 30%, GU 12.9%, Lung 4%	Khorana Score ≥ 2 Prior VTE Metastatic disease Macroscopic vascular/ lymphatic compression	1 1 1	0 (Low) 1 (Intermediate) ≥2 (High	Ext. Validation: 0.59 [79], 0.89 [80]
COMPASS CAT	Breast 61.5%, Colon 16.6%, Lung 13.3%, Ovarian 8.6%	Platelets ≥ 350 G/L Prior VTE ≤6 months since cancer diagnosis Antihormonal therapy in HR + -breast cancer Anthracycline chemotherapy Central venous catheter Advanced/Metastatic disease Cardiovascular risk factor Recent hospitalization/acute medical illness	2 1 4 6 6 3 2 5 5	0–1 (Very Low) 2 (Low) 3–4 (Moderate) ≥5 (High)	Derivative: 0.85 Ext. Validation: 0.62 [81]
Tic-ONCO	Colon 41.7%, Pancreas 18.4%, Lung 22.3%, Esophagus 3.6%, Stomach 14.1%	Very high-risk tumor types High-risk tumor types Genetic risk score based on SNPs BMI > 25 kg/m^2^ Family history of VTE Tumor stage	X X X X X X		Derivative: 0.73
Ottawa Score	Lung 17.7%, Breast 15.6%, Gastrointestinal 25.8%, Hematologic 10.7%, Other 30.7%	Female Sex Lung Cancer History of VTE Breast Cancer Cancer Stage I and II	1 1 1 −1 −2	≤−1 (Low) 0 (Intermediate) ≥1 (high)	Ext. Validation: 0.60 [68]
SAVED-Score	Multiple Myeloma (IMiD)	Prior Surgery Asian Ethnicity VTE history Age ≥ 80 years old Dexamethasone Standard dose (120–160 mg) Dexamethasone high dose (>160 mg)	2 −3 3 1 1 2	≥2 (High risk)	Derivative: 0.61 Ext. Validation: 0.60 [70,72]
IMPEDE VTE Score	Multiple Myeloma	Immunomodulatory agent BMI > 25 kg/m^2^ Pelvic, hip or femur fracture Erythropoietin stimulating agent Doxorubicin Dexamethasone Low-Dose Dexamethasone High-Dose Asian Ethnicity VTE history Central venous catheter Existing thromboprophylaxis (Therapeutic dose) Existing thromboprophylaxis (Prophylactic dose)	3 1 2 1 2 2 4 −3 3 2 −5	≤3 (Low) 4–7 (Intermediate) ≥8 (High)	Derivative: 0.66 Ext. Validation: 0.62 [72]

Abbreviations: BMI: body mass index; ECOG: Eastern Cooperative Oncology Group; ESA: erythropoiesis-stimulating agent; Hb: hemoglobin; SNPs: single-nucleotide polymorphisms; VTE: venous thromboembolism; WBC: white blood cells.

**Table 2:** Examples of cancer risk and indications for primary prevention.

**Risk for VTE: Cancer type**	
**Cancer: Examples**	**Risk for VTE**
Gastric, pancreatic, esophagus, biliary	Very High
Non-Hodgkin lymphoma, lung, genitourinary cancer, uterus, ovary, brain, sarcoma, myeloma	High
Colorectal	Intermediate
Lymphoblastic leukemia, other cancers	Low
**Risk for VTE, Patients with cancer: Race**	
**Race**	**Risk for VTE**
Black	Highest
White	Intermediate
Hispanic	Lowest
Asian	Lowest
**Risk for VTE: Patient’s age, gender, morbidities**	
**Patient’s characteristics**	**Risk for VTE**
Older patients, men, chronic illness	Greater
Younger patients, women, healthy	Less
**Indications for Primary Prevention of VTE**	
**Khorana score ≥ 2**	
Myeloma patients receiving immunomodulatory drugs	
Certain cancer types such as advanced or metastatic pancreatic cancer	
Hospitalization, especially with medical illness	
Surgical interventions	
**Appropriate Drug Regimens for Primary Prevention of VTE**	
**Patient Groups**	**Drug Regimen**
Ambulatory cancer patient	Low molecular weight heparins Direct oral anticoagulants
Hospitalized cancer patients	Low molecular weight heparins preferred
Surgical cancer patients	Low molecular weight heparins or unfractionated heparin Rivaroxaban or Apixaban (extended duration anticoagulation in high-risk patients)

**Table 3:** Anticoagulant options for cancer-associated venous thromboembolism by treatment phase.

Treatment Phase	Recommended Anticoagulants	Standard Dose Regimen
Initial treatment (first 5–10 days or longer)	LMWH (enoxaparin, dalteparin, tinzaparin); UFH; fondaparinux; DOACs (apixaban, rivaroxaban, edoxaban *)	Therapeutic anticoagulation using weight-based LMWH, intravenous UFH, weight-adjusted fondaparinux (5 mg < 50 kg, 7.5 mg 50–100 kg, 10 mg >100 kg subcutaneously once daily), or DOACs initiated with standard loading regimens
Early maintenance (up to 3–6 months)	LMWH or DOACs (preferred); VKA (alternative)	LMWH or DOACs (preferred); VKA (alternative)
Extended treatment (>6–12 months, active cancer)	LMWH preferred; DOACs used with caution (especially GI/GU cancers)	Continued full-dose anticoagulation
Extended secondary prophylaxis (>6 months, selected patients)	Apixaban or LMWH	Apixaban at full dose (5 mg twice daily) or reduced dose (2.5 mg twice daily), based on bleeding risk
Long-term therapy (beyond 12 months)	Individualized (LMWH or reduced-dose DOAC)	Risk-adapted anticoagulation, with reduced-dose apixaban favored in selected patients

* Edoxaban requires ≥5 days of parenteral anticoagulation before initiation.

## Data Availability

No new data were created or analyzed in this study. Data sharing is not applicable to this article.
